# Valedictory Address

**Published:** 1841

**Authors:** Thos. E. Bond

**Affiliations:** Professor of Special Pathology and Therapeutics.


THE AMERICAN JOURNAL

OF

### DENTAL SCIENCE.

VOL. I.—NOS. XI & XII.

[aug. + Sept. 1841.]

### VALEDICTORY ADDRESS

*To the Graduates of the Baltimore College of Dental Surgery, delivered at the commencement, March 9th,* 1841---BY THOS. E. BOND, JR., M. D. *Professor of Special Pathology and Therapeutics.*

PUBLISHED BY REQUEST.

After the diplomas had been presented by Professor Hayden, Professor Bond arose and said,—

GENTLEMEN,

The time has come when the relation of teacher and pupil, which has for some time existed between us, must be dissolved, and in the name of your preceptors I am about to bid you farewell.

It affords me no little pleasure to believe that our intercourse has been mutually agreeable and beneficial; and that neither you nor we will regret the time and labour of the past session.

You are aware that the Baltimore College of Dental Surgery went into operation under very discouraging circumstances. The difficulty of the undertaking is abundantly proved by the remarkable fact that, notwithstanding the necessity of scientific education in Dental Surgery had long been felt, and the difficulty of procuring it bitterly lamented, no attempt had been made to afford collegiate opportunities to pupils in the art, until the faculty of the present school risked the adventure. It is unnecessary now to enter upon a minute detail of the causes which rendered success improbable. Suffice it to say that the projectors of the school had abundant reason to anticipate open hostility from many, and secret enmity from more. For this, however, they cared but little; but they feared that which is far worse than opposition, the entire indifference of the community to the result of the enterprize.

He who pleads the cause of truth may convert an enemy, and, perchance make him his friend ; but even truth is impotent where it is not heard. Opposition gives warmth and energy to the spirit ; even persecution, as it cannot subdue the soul, may rouse and elevate it ; but indifference presents nothing upon which the mind can act. It has neither incitement for the present, nor hope for the future. It is a very dead sea amidst whose leaden waters the mind may wear out its strength without effect, or even pleasure in the struggle.

To fortify us against these fears of the public neglect, we of course had the common consolation of all who labour for the multitude, viz : the comfortable assurance that if we *would* go on, the public would sustain us. It is to be regretted that those who are most ready to make this pledge for the community are generally such as know but little of the philosophy of public action ; and it is more lamentable that many believe to their sorrow, promises so easily made and so seldom realized. The fact is, the great public, with all deference to multitudinous majesty be it spoken, are much more prone to follow effort than to lead and sustain it. They are, in fact, little better than the campfollowers of enterprize, ever ready to join in the huzzahs of victory for which they have not fought, and to share the spoil they have not won ; and just as ready, should the result be different, to embarrass and insult a defeat they ought to have prevented. From the public, then, we were taught by experience to expect much the same treatment as that they bestowed upon the Vicar of Wakefield's philosophic son George. You may remember that this poor young man, relating his adventures to his father, informs him that to attract some attention, he dressed up three paradoxes with some ingenuity, and published them. "I made no doubt," he says, "that the whole learned world would oppose my systems ; but then I was prepared to oppose the whole learned world." "Well, my son," said the good old Vicar, "and what did the learned world say to your paradoxes ?"—"Sir, replied George, "the learned world said *nothing* to my paradoxes : every one was employed in praising his friends and himself, and condemning his enemies ; and as I unfortunately had neither the one nor the other, I suffered neglect."

To sustain us against the opposition of our enemies, and the terrible vis inertiæ of the public mind, we relied chiefly upon the support of those Dental Surgeons whose approbation is most honourable, and whose influence is most efficient. In this we have not been disappointed.—On the contrary, we have been encouraged, and cheered on by the countenance and sympathy of the most skilful and distinguished of the profession on both sides of the Atlantic. To the medical profession, too, we are happy to acknowledge ourselves under great obligations. From the first, they have been our warm and zealous friends. They have despised the fitful jealousy, so often and so falsely attributed to them, and every where and in every way, they have promoted the success of an infant enterprize. It does not become me to sound the praises of the medical profession; but it is a high minded and generous calling proper for highminded and generous men; and I rejoice to know that many such everywhere swell its honourable ranks.

But amidst the anxiety of a first session, nothing has been so cheering to us as your untiring industry, and the evidence you gave of rapid advancement in knowledge. In the name of the Faculty, I thank you for the comfort and encouragement your deportment has given us; and I sincerely wish, that if at any time either of you should become a teacher, you may have just such pupils as you have been.

Your appearance here to receive from us the strongest testimonial of professional qualification that has ever been bestowed upon a Dental Surgeon, is sufficient evidence that you have not attended in vain to the instructions we have endeavoured to impart. After having admitted you to the honor of a degree—an honor the dispensation of which has been committed to us by the State of Maryland, and which we are bound in good faith to bestow upon none but those who deserve it, it would be supererogatory to expatiate upon the confidence we have in your ability to give credit to the institution that presents you to the world as its first fruits. We can add nothing to the solemnity of the act by which we have recommended you to the confidence of the community; nor indeed is any additional commendation necessary. Wherever you go, whether in Europe or America, the diploma you hold in your hands will be a passport to public consideration, and an abundant introduction to usefulness. You have been taught that Dental Surgery is not a mere art separate from, and independent of, general medicine; but that it is an important branch of the science of cure. Inquiring into the diseases of the antrum alone was sufficient to convince you that the teeth and their dependencies are often primarily interested in the production and aggravation of some of the most fearful and fatal of human disorders; and you have at once been compelled to discard the miserable fallacy, that the teeth are mere supernumerary organs, not at all concerned in the general health, and capable of being maltreated at the pleasure of ignorance, without inducing serious results.

Your knowledge has been based upon extensive and accurate anatomical investigation. You have seen and traced out the exquisitely beautiful machinery by which the organism is every where knit together. You have learned the secrets of nervous communication, and studied the simple yet admirable arrangement by which nutrition is drawn by each part from the common receptacle of strength. You have also carefully examined the phenomena of health and disease, both as they are manifested in the dental arch, its connexions and relations. Your attention has been particularly directed to the effect of local irritation upon the general health, and you have seen how readily organs apparently unconnected, and independent may be involved in mutual disease. You have been taught to regard the human body as one complete whole, united in all its parts, and pervaded every where by strong and active sympathies; and your principles of practice have been carefully formed upon a sound knowledge of general medicine.

I trust, gentlemen, that in the course of enquiry in which we have been employed together, we have learned something more than mere physical truths. We have been busied in examining the most equisitely beautiful and wonderfully perfect of all machines: we have wondered at the skilful contrivance by which various complex and highly delicate organs endowed with exquisite sensibility, and liable to disorder and decay, and bound up together so compactly as to occupy the least possible space, and yet so securely as to work on in their several positions for years without pain or injury,—notwithstanding the careless and violent motions of the body. We have observed how perfectly, as by some intuitive power of choice each organ performs its own appropriate work, none ever mistaking its duty. The same blood is sent to the different secreting organs, and by an admirable and inscrutable arrangement each separates from it the substance peculiarly its province to take away, at the same time that all are nourished and sustained by it. A perfect understanding seems to exist among the different parts of the body, which enables them to move on harmoniously in their closely connected, yet separate labour. These and a hundred other admirable and inexplicable marks of infinite power and wisdom, we have been privileged to observe and reflect upon. Can it be that we have admired the workmanship and thought not of the Almighty and all-glorious workman? Have we trembled to behold how wonderfully and fearfully we are made, and remembered not the Being upon whose will alone our life depends?—The heavens declare the glory of God and the firmament sheweth—His handy-work! but these glorious works are not worthy to be compared with the mysterious fabric which is at once the dwelling place and the instrument of the spirit of man! They are inert, unconscious matter: the body of man is endowed with life, and with the grand and effective power of will! Nor do the glorious sun and burning stars teach all the great truths which may be read in the minutest tissues of the human frame. It is indeed delightful to raise our eyes from the earth to those pure creations of God, which only of all His visible universe remain unsullied by sin. There they shine forth in the beauty of their original brightness, enlightening the world their undimmed radiance condemns,[as though they would at once reproach the guilty earth and woo it back to purity. It is well to gaze upon them, for God has hung them out in the heavens to light up the approach to his throne. From age to age they testify of His holiness, and His power. But alas! they tell of endurance, of unfading glory, and man needs lessons of decay and death. They witness the revealed truth of creation; but man needs to be confirmed in the dreadful revelation of the fall. Let him then study the striking combination of this appalling truth as he finds it recorded in the condition of his own body. Here the philosopher will find the striking anomaly of a perfect work fully adequate to its ends; and yet full of imperfection in its operations. He will see, too, a body dying from birth; yet sustained by an unseen power through the various stages of life. The human frame as it presents itself to our observation, is an inexplicable riddle—a bundle of inconsistencies until explained by the Holy Book. When we survey the perfection of its plan, and the exact management for the performance of every part of its details, and then observe that there is superadded to this, an every-where watchful and powerful ability to remove difficulty and repair damage, we might naturally suppose the machine to be indestructible ; but when, made aware of its liability to disease and destruction, we remark the extreme delicacy of its tissues, and learn how very slender is the membrane or ligament that intervenes between life and death, we are again lost in wonder, that a machine so complex and delicate, and prone in every part to dissolution, can be made to survive the rough usage of life for an hour. In a word, the human body is a standing witness to the truth that it was once perfect, and subsequently has been blighted. Nothing but the power of the omnipotent God could have created it, and nothing short of the same Almighty agency could remand it to the dust. If the study of anatomy thus teaches the doctrine of the creation and the fall, its physiology and pathology abound with evidences of the benevolence and mercy of God.

To the observing eye, it is every where evident that the Great Being has tempered his justice with mercy. If He has condemned us to death, He has made provision for length of life abundantly sufficient for the purposes of temporary existence, and sufficiently short for the patient endurance of its sufferings. If He has cursed the body of man with the necessity of pain, He has distributed sensibility so benevolently that pain itself is become a blessing without which life would be a succession of horrors and alarms. He has made it an unwearied and faithful sentinel upon the outposts of life ; ever ready to give warning of danger, or to summon the reparative powers to the aid of a wounded or suffering organ. The mother who sighs over her infant writhing in pain seldom thinks how much both she and her child are indebted to its power of suffering.

Yet without it, disease might have destroyed the unconscious babe before maternal vigilance could have detected the existence of disease. The pain she deplores is the watchman more vigilant than she, that gave the alarm in time to repel the danger. The most watchful nurse unconsciously abandons her helpless charge to the sleepless fidelity of this friendly yet harsh protector, and slumbers with perfect security by the side of her infant, well assured that at the first approach of want or danger, she will be roused to its assistance.

The sense of pain has not been capriciously interwoven with our organism. On the contrary, it has been most carefully located with evident reference to our convenience and our safety. The skin which covers the whole body, and is of course exposed to the first impressions of external agents, is endowed with the greatest sensibility. It is the first great outpost to the system, and is therefore necessarily intrusted with the power to distinguish the slightest approach of evil, and to send the alarm to the inmost recesses of the body. But, contrary to the common opinion, the deeper an organ lies, and consequently the less exposed it is, the less is its sensibility to pain ; and the bones which have nothing to fear from external injuries, except such as must first spend their force upon the sensitive organs that envelop them, have scarcely any sensibility at all.— Are these not evidences of wisdom, of benevolence, of mercy ? Do they not loudly reiterate the doctrine of revelation : — that God has cursed, and yet loves the body of man ? Will we be told that the superior sensibility of the skin, is a mere accident of formative power, or that it results from the peculiar exposure of that organ to exciting and irritating causes ? Facts disperse the supposition, even if it were possible to conceive of any other formative power than God. The location of pain has evidently not been determined upon any general rule. When any part of the integuments is of a character that would render sensible a most fearful evil, sensibility is witheld. The hair, for instance, has no sensibility. Is this a mere accident ? Think for one moment if you can, of the dreadful condition to which man would be reduced by the transfer to the hair of the sensibility of the skin. It would of course grow to a great length. Every fibre of the mass would soon become irritated and inflamed and the seat of agony. It would be impossible to remove it without a most painful surgical operation. And yet life would be insufferable if men were compelled to drag behind them a tangled mass of disease which filled the whole frame with exquisite agony. So we might remark of the nails. The apparently trifling provision of God by which these are made insensible, is in truth a provision absolutely necessary to the smallest degree of comfort to man. It has often been said that the study of medicine inclines men to infidelity. I am sure this is not the case. On the contrary, I am happy to believe that there are comparatively very few in our profession who do not believe in God and in his word. It is true that the study of science will not necessarily prevent a man from disbelieving—for pride, which is the seed of scepticism, is able so to stultify the mind that it becomes impenetrable to evidence.— But where a student of medical science does become so lamentably stupid as to be unable to realize the being and the character of God, depend upon it his daik condition has been brought about not through medical knowledge, but in spite of it.

I am aware that French and German atheism has laboured hard to make Anatomy, as well as other branches of science, instrumental in sustaining and propagating its silly and heathenish notions. And never has ignorance made more awkward and ridiculous efforts to appear wiser than when gravely philosophizing upon the human body.

If there is anything more sagely fantastical than another, it is sceptical transcendental anatomy. Folly here, seems more than commonly foolish ; and as Cicero said of the Roman augurs, we may well say of the transcendental anatomists, that it is strange how two of them can look at each other without laughing. Their theories exhibit little else than learned stupidity decked out in the robes of science.

Time will not permit me to dwell longer upon this subject. So far as you are concerned, I am sure that no argument is necessary to convince you that medical science nowhere contradicts divine revelation ; but on the contrary every where confirms and proclaims the truth of the Holy Book ; and I trust that there are none in this audience so unfortunate as to have yielded credence to the smatterers in knowledge, who find upon the very surface of speculation what seems to them conclusive evidence that christianity is a fable, and all the wise and good who profess it, either charlatans or fools.

I trust that our confidence in our faith is too well grounded to be shaken by the clownish attempts of physiologists to explain away the necessity of a creator; or by the crude and illogical conclusions of the geologist who refutes Moses by an essay upon oystershells ; or yet by the deistical theologian who assures you, with great gravity, that a miracle is altogether unnatural, and very rationally explains the angelic annunciation of the birth of Christ to the shepherds, by supposing that they were humbugged by a wandering Bethlemite with a lantern !

Truly, gentlemen, a little knowledge is a dangerous thing—and moreover it is a very presumptuous and noisy thing. The character of modern infidelity, whether anatomical, geological or theological, may be written in three words, '*Vox et prœterea, nihil !*'

Gentlemen, you are now members of a useful and honorable profession. You go forth to the world with high testimonials of character and skill; and we have a right to expect from you such conduct as will reflect honor upon us and upon the profession you represent. A greater than ordinary responsibility rests upon you. Under any circumstances the honor and dignity of any profession is intimately connected with the deportment of each of its members; and that man is a traitor to his brethren, and a libeller of his class, who by his negligence or vice, degrades the pursuit or the profession in which he is engaged.

But the circumstances under which you commence your career are of rare occurrence, and involve you in responsibility which seldom falls to the share of two young men. This day, for the first time in the history of the world the practice of dentistry is legally recognized as a profession, and you are the first who are permitted by public authority to be distinguished by the title of Doctor of Dental Surgery. As soon as you go out in the world, the very title you bear will subject you to scrutiny, as close as jealousy and envy can institute. Your conduct will be subjected to the most microscopic investigation, and your errors magnified and multiplied by all the appliances of scandal. You will require more than ordinary virtue and circumspection to enable you to sustain the trial, but should you fail under it you may inflict irremediable injury upon the profession you have chosen, and which is now struggling for a dignified position in the public estimation. Let me beg then that from the very first you will assume a dignified stand, and that you will religiously maintain it. Arm yourselves with a fixed determination to do honour to the title you bear—to give it honour in the eyes of all men, and to force even the most invidious to respect it.

Your titular distinction is founded upon admitted superiority of knowledge ; but you must remember that it will require continual diligence to maintain that superiority. The science of medicine is ever improving—hundreds of active and intelligent minds will soon be devoted to the improvement of that branch of the healing art to which your attention will be chiefly directed; and unless you continually add to your present stock of information, you will soon find yourselves distanced by those who are now but tyros in knowledge compared with you. The expression—"I have finished my education," does well enough for the finished gentleman and gentlewoman in their teens, who have learned science by questions and answers in boarding schools; but it is the height of folly for the *student* to talk of having learned enough. To him, every acquisition in knowledge is but the threshold of higher attainments; and when he is looked upon by others as wise, he feels that he has learned but the rudiments of wisdom. Be diligent, then, both to acquire information, and to impart it; feeling it to be your bounden duty, as well as your pleasure, to contribute what you can to the improvement and dignity of the profession you have assumed.

But, gentlemen, learning alone will neither make you extensively useful nor respectable. A notion has some how or other got footing in the world that superior knowledge is most respected when associated with rude and vulgar manners; and as the latter supposed qualifications of genius are much more easily acquired than the former, we are abundantly supplied with professional bruinism. Now, gentlemen, this notion is sheer nonsense. Dr. Johnson would have been quite as learned, and far more respectable if he had been a gentleman; and Mr. Abernethy would have been quite as eminent a surgeon, and a far more useful man, if he had not studied clownish deportment. I know that it is common to call the rudeness of the learned by the milder name of eccentricity; and many who are not learned would fain be odd, and enjoy the enviable distinction of doing things which gentlemen do not generally do. Now, gentlemen, when I hear a man spoken of as eccentric, I either pity him, or despise him, as I have reason to believe his deviations from propriety to be the result of nature, or of study. If they are not natural, the man is insane precisely to the extent that he is odd; and generally his eccentricity ends in serious aberration of mind: of course I pity him as a sufferer from this most fearful of afflictions. If he is not mad, I cannot but despise him for being such a fool as to take pains to pursuade people that he has less than common sense.

Depend upon it, gentlemen, that a polite and benevolent deportment is becoming to all men, and grateful to all men. Indeed, it is the duty of every man to be kind and courteous to all with whom he has to do; and *he* is altogether inexcusable who is habitually impolite. By politeness, however, I do not mean obedience to certain conventional rules which regulate the intercourse of the frivolous and the fashionable; but I mean that active benevolence which strives always to create as much comfort and happiness as possible, and thinks no sacrifice too great, and no attention too small, which may contribute to this end. It is highly necessary for your own happiness and the comfort of others, that you cultivate this spirit. And I would especially beseech you to be careful to exercise it towards those practicing dentists who have not enjoyed similar advantages with those you have had. Never presume upon your superior acquirements, so as to lead you to treat them with disrespect, unless you have reason to believe that they are mere empirics who are abasing the confidence of the public. To all worthy men, whatever be their attainments, who are engaged in the practice of dentistry, behave with kindness. Give them what instruction you can. Try to stimulate them to acquire more knowledge, than either you or they possess. Give them credit for all that is commendable in their practice, and be silent to all but themselves about any errors they may have committed. Let it be a rule of your life ; and endeavor to make it the disposition of your hearts to speak evil of no man—except in such case as you are well convinced that greater evil would result from your forbearance.

You will doubtless meet with many instances of flagrant imposture ; and you will be vexed by the success of ignorance and impudence in deceiving and robbing the credulous and the unsuspecting. In such cases your duty is plain. Whenever you can disabuse the public mind, or prevent injury to an individual you should not hesitate for a moment to do so, without reference to the consequences to yourself. But where you have no reason to believe that your interference would prevent mischief, let it be impressed upon your minds that it is no part of your duty to make war upon scoundrels. You are not expected to be public executioners. To chastise roguery and to expose ignorance wherever you may meet it, would require the hundred hands of Briareus and the industry and strength of Hercules. I know that the success of quackery and effrontery is trying to the spirit of an honest man ; but I have learned that nothing is to be gained by fretting about that which we cannot help. Men have been cheated by quacks and charlatans from time immemorial, and will be so cheated after you and I have ceased to observe it. The passion for nostrums is too strong for reason. You could as easily knock down a pyramid with your fist as to prevent people from swallowing Pease's Candy, or having their teeth filled with mineral amalgams. In a former lecture, at which you were not present, I took occasion to dwell at some length upon what might be termed the philosophy of empiricism, and endeavoured to show that it was inseparable from the present condition of the public mind.

The success of quackery in our land is indeed one of those strange things that, at first sight, appear to baffle philosophy, and to present to the curious investigator the anomaly of a thing done in contrariety to apparently well known circumstances that should have prevented it.

We have been in the habit of supposing that empiricism is but one mode by which the cunning prey upon the simple ; and that its success must be proportionate to the ignorance and mental inactivity of the people upon whom its arts are practiced. This being the case, we are astonished to find that quackery of the grossest character feeds and fattens upon the American people, whom we reasonably believe to be the best informed and the most thoughtful of all the communities of the world.—Moreover, although the fact is well ascertained that the general intelligence is progressive, yet imposture seems to grow with the growth of mind.

These facts are the natural and philosophical results of something.—There is reason for them, and upon reflection upon these things, we may learn some unexpected truths in the philosophy of mind.

Perhaps the true way of getting knowledge is to reflect upon every thing that we do not fully understand. By investigating thoroughly even the follies of mankind, we may acquire much correct information concerning human nature, and our own nature, as human.

The history of quackery, whenever it shall be written, will be exceedingly entertaining and instructive. It is remarkable that as far as we have any knowledge, empiricism has been practiced in all ages upon precisely the same principles, and with much the same results, as it is at present. There must be a charm in taking physic, which only the takers know, for the Arabians and the Greeks have opened their mouths to much the same drugs and drenches that are swallowed with such gusto by the modern patrons of vegetable pills and health-preserving potions.

By tracing the history of empiricism, we find that it has always been of two kinds, founded upon two different characters of people among whom it prevailed.

There are two distinct tendencies in the human mind which are opposed to one another, and yet may be made to produce much the same results. These are—*Superstition and Credulity.*

Dr. Paris has defined these two principles thus: "*Credulity* is an unbounded belief in what is possible, although destitute of proof, and perhaps of probability. *Superstition* is a belief in what is wholly repugnant to the laws of the physical and moral world."

Now, in times of gross ignorance, men are always *superstitious;* but they are so far from being *credulous,* that it is difficult to obtain their assent to the result of any process of clear reasoning, if that result be different from their previous notions. On the other hand, when intelligence is widely diffused, and the mind is worked up to enquiry, *superstition* loses its hold. Men are apt to refuse assent to every thing that does not profess to be the result of human research; but at the same time they are abundantly ready to believe the most absurd propositions if they present themselves as the fruit of experiment, or the result of human effort. In short, while they have no faith in the production of ends by means not understood, they have unlimited confidence in the power of means to bring about ends the most extraordinary.

*Credulity,* therefore, waxes, as *Superstition* wanes.

Thus the rulers of the French nation, after having thrown off the shackles of superstition with such mad violence, as to tear away with them all reasonable faith in the supernatural, became so exceedingly credulous, that they were readily imposed upon by the extremely stupid fooleries of Anarcharsis Clooty, the embassador of the human race.

The same experience that leads men to throw off all faith in pretended miracles, makes them acquainted with so many extraordinary results, developed by the power of mind, that they do not know how to limit their expectations of success in the various branches of human pursuit.—Being often disappointed, by the performance of things they had thought impossible, they cease to reason upon probabilities, and learn to think all things possible.

Together with this state of the mind, there grows up a fondness for novelty, which being continually fed, becomes imperious and seizes with eagerness upon whatever food may be presented to it.

Let us illustrate these remarks:

The American people are not *superstitious.* The Africans, though superstitious, are not *credulous.* Yet the former are as readily deceived as the latter. You could not persuade the American people that a man's eyes had been miraculously opened to behold the inhabitants of the moon; but it would require little effort for an Obi-man to persuade the savage African of this. On the other hand, you could not make the African believe that a man had invented an instrument, without supernatural assistance, by means of which he could see the inhabitants of the moon; yet a large portion of the American public actually did believe this, upon no better authority than the assertion of an anonymous newspaper writer. An American would not believe that a physician could make a charm to prevent disease; an African could not be persuaded that cold is the cause of all diseases; or that the activity of medicines is proportioned to the diminution of the dose. The American would not wear a fetish; the African would not trust himself in the steam-bath of a Thomsonian, and would laugh at the solemn fooleries of Homœpathy.

In short, the difference between us and the savage, with regard to imposture is, that we require to be cheated into a belief of the wonderful; while the African, having no faith for what is merely extraordinary, offers no resistance to the reception of the marvellous.

Now, quackery has always taken the color of the age in which it prevailed, and has appealed to *superstition* or *credulity*, accordingly as one or the other was paramount in the public mind. So true is this, that were other items of history absent, we might form some correct opinions of the character of a community from a knowledge of its empiricism.

Rhazes, a celebrated Arabian physician, has left a description of the quacks of his day, which shows clearly that the Arabians were then a credulous rather than a superstitious people; and by inference we may safely conclude that they were to some extent, intelligent and active.—Indeed, they were at that time the most enlightened and learned people in the world: and having thrown off the shackles of Paganism, were but little inclined to superstition. Yet they had their share of quacks, who seem to have been very ingenious in their impostures, much more so, indeed, than are some who gull us so easily. Some of them professed to cure epilepsy by pretending to make an opening in the posterior part of the skull and removing something from the brain. Others cured certain disorders by extracting little lizards from the nose. This they effected by dexterously inserting an artificial animal, and then withdrawing it. Others extracted little frogs from beneath the tongue, which they managed in a similar way. Others, again, attributed their patients' maladies to the presence of glass, or other foreign bodies, which they had swallowed, and which, in fact, the quacks pushed down the throat while pretending to examine it.

All this may appear to us very silly ; but it required more dexterity and talent to impose upon people in this way, than ever was manifested by the stupid, yet successful empiric, Sweet, who told all his patients that their hips were dislocated, and pretended to replace the bone in its socket by a gentle manipulation causing no pain or scarcely any sensation to the subjects of the operation.

You will perceive that all the modes of imposture related by Rhazes, were addressed to the credulity of the people.

While these impostures were practiced in Arabia, the Europeans were sunk in gross darkness and ignorance, and of course were the slaves of Superstition. Quackery abounded among them too, but it addressed itself, not to their credulity, but to their superstition. Indeed, medical science was overgrown, and almost lost amidst the luxuriant growth of empiricism.

Experience and philosophy were no longer the bases of practice ; witches and wizards, and the monks were the doctors, and charms and amulets, and holy water formed the remedial agents. The competition between medical theories for the greater part, narrowed itself down to the rival claims of good and evil spirits ; and the wise no longer disputing about the nature of disease, transferred their secreting to the supernatural agency by which it was brought about. For some time, there was a hard contest between the devilists and monks ; but the latter appear to have been outnumbered, and Satan established his supremacy as a quack, over all opponents.

Godelman, Cardan, Arterius and others, contend that Satan was the best physician, upon the ground, we suppose, that as he created diseases, he knew best how to cure them. But some did not confine his skill to such disorders as he and his imps had made. Nicholas Taurellus says,—"Many doubt whether the devil can cure such diseases as he hath not made ; and others fully deny it : however, *common experience* (see how naturally all empiricism appeals to experience for support against common sense) confirms, to our astonishment, that magicians can work such feats, and that the devil can, without impediment, penetrate through all the parts of our body, and cure such maladies, by means to us unknown."

The fact of the devil's skill, being thus fully ascertained, like the powers of quack medicine in our day, by experiment, the other party raised a question, whether it was lawful to apply to the evil one for relief, when the saints were too busy or were unwilling to grant it. Various divines and schoolmen denounced Satan's practice as abominable, and declared that it was better to die than be cured by him. Paracelsus, however, and doubtless many other utilitarians, agreed rather impiously, that it mattered very little to a man, whether he were cured by angels, or by unclean spirits, so that he were cured. He says,—"If a man fall into a ditch, what matter is it whether a friend or any enemy help him out ? If I be troubled with a malady, what care I whether the devil himself, or any of his ministers redeem me ?" piously adding, however, that these things could only be done by God's permission. Paracelsus therefore recommends incantations for the removal of disease, while various divines ran an opposition line of adjuration and exorcism, by fire, suffumigations, lights, cutting the air with swords, sacred herbs, odours, &c.

Some of the remedies got up by quacks and extensively used at that time, are almost too absurd for belief. A ring made of the hinge of a coffin, cured cramps. Nails driven in an oak tree cured tooth-ache. A halter that had served for hanging a criminal was an infallible remedy for head-ache, if tied around the head, or better if beaten to powder and used as a snuff. The chips of a gallows tied up in a bag, and worn round the neck, was famous for the cure of ague, and a stone with a hole in it was infallible to prevent witches from riding men and horses.

It is remarkable that some of the superstitious practices that arose in the dark ages, are still continued among us without any reference to the original design for which they were introduced. Coral, for instance, was once considered very powerful in keeping off the evil eye, and scaring mischievous spirits. For this reason it was worn as an amulet; and Paracelsus tells us that it should be constantly hung around the neck of infants to preserve them against fits, charms, &c. It is still used for necklaces and toys for infants, though few are aware of the important advantages the little things derive from it.

When letters began to revive, and superstition to decline, empiricism took the color of the age, and credulity was appealed to by ingenious quacks.

There is one case upon record, however, which appears to be a sample of abnormal quackery, for the impostor founded his expectations of success, neither upon the prevalence of superstition nor credulity, but simply upon the fears of the people.

In the year 1760, a mad soldier of the guards, in London, predicted a terrible earthquake, upon which a bold speculator in panics sold a vast quantity of pills as being good for earthquakes! So says the record,

> "I know not what the truth may be,
> But tell the tale as told to me."

Dr. Tissot, a Swiss physician, in a work pulished in 1760, has an excellent chapter upon empiricism from which we learn, that among that intelligent people, the same sort of quackery prevailed, as prevails here at this day; viz: the quackery of credulity. His description of empirics might be stereotyped for all ages. He says "They are constantly vile wretches, who incapable (or unwilling) to make a livelihood in any honest way, have laid the foundation of their subsistence, upon their own amazing stock of impudence, and the weak credulity of the people. They have no scientific knowledge; their certificates of cures are so many chimeras or forgeries, and in short, if among the prodigious multitudes of persons who take their medicines, some of them should recover, which it is almost physically impossible should not sometimes be the case, yet it would not be the less certain, that they are a pernicious set of men. A thrust of a rapier into the breast, has sometimes saved a man's life, by seasonbly opening an abscess, yet wounds with a small sword, are not desirable on this account."

In our own time and in this community, empiricism is as successfully resorted to, as it has ever been in any age or among any people.

There are several classes of the community, upon whose patronage the quack may always reckon. The first consists of that miserable class, who live in perpetual dread, and daily die a thousand deaths in fearing one!

These poor people, are ever imagining that they feel within them, the first tremblings of dissolution, and their confidence in the powers of medicine is generally in increased proportion to their confidence in their own vitality. These are like the horse-leech, ever crying "give !!" Their capacity is boundless. Potions and pills, pastes and panaceas, all find their throats an open highway. Their ability to receive, cannot by any possibility, be exhausted, for they feed a phantom. Like the man who imagined he had a wolf within him, that could never be satisfied with food, these unhappy creatures have an imaginary inmate that is insatiable.

There is another class who are too wise to be cured by ordinary means. There are many, generally persons of weak minds, who have a raging desire to be doctors, and as they are excluded from the rank of the regular profession, either by their sex or by their ignorance, they naturally become at once the apostles, and the prey of quacks.

There is another class, unfortunately always a large one, who labour under hopeless disorders, and are anxious to try every thing that promises relief. These are sincerely to be pitied. They are the severest sufferers from empiricism.

There is still another class, though perhaps a small one, who are absolutely amateurs of physic. They are neither driven to it by imaginary disease, nor by real suffering, but they seem to take it by way of amusement. I know some that regularly take all the new physic that comes out. Thomsonianism and Homœpathy, have equal charms for them, and Dutch doctors, and Indian doctors, are blessed by their equal confidence.

I have spoken of the present as a credulous age. I now add that it is not a *wise* one—with all our pretensions to intelligence we are not a *wise* people. The history of every day proves this. We live in an age of *talent*, and we are a *talented* people. This is our characteristic, and not wisdom. There is a great difference between talent and understanding. Understanding *conceives* a plan, *talent executes* it. Talent is the efficiency of a man; the power to perform what he wills. Now, we have this power in an eminent degree ; but as a people, we do not think, and reason, and determine correctly. The whole energies of the people are directed to increase and develop talent, not to gain wisdom, to multiply the powers of action rather than to establish sound principles whereby to direct them. This indeed is the condition of the whole enlightened world; and it grows out of the fact that in the present state of the human mind, men find it easier to study what is external and visible than what is metaphysical.

We regard the present as a transition age—the age of talent, yet to be followed by the age of truth.

There are certain usages established among medical men, which are highly honorable, and indeed, absolutely necessary to the preservation of the dignity of the profession. The first that we shall mention, has reference to their treatment of one another, and is commonly styled medical etiquette. Now this etiquette is something very commonly misunderstood by the public. They seem to think that it is a formal code of quixotic regulations, altogether unnecessary, and very troublesome to patients, and there are not wanting men who bear the honorable title of physician, who for the advancement of their own little ends favor this opinion.—Now, gentlemen, the truth is that just so far as a man is honourable and upright, he will be a strict observer of these rules, and you may lay it down as a rule, that a physician who disregards or rails at medical etiquette is a mean, selfish, skulking fellow, who in his heart neither fears God nor regards man. This expression may seem harsh, but it is true, for medical etiquette is nothing more than professional observance of the golden rule, 'do unto others as ye would that they should do unto you.' It teaches that physicians are brethren, equally exposed to the many unpleasant things connected with a most anxious, laborious and often ill requited profession; equally subject to caprice, to unjust suspicion, and to unmerited censure, and hence bound together by sympathy which they can look for no where else. It bids them to sustain each other in the time of trial; to bear one another's burdens; to make the reputation of each other the care of all; and thus in every possible way to smooth the thorny road of professional life. It is strange that the public cannot see the beauty and propriety of these regulations; but I trust that you can see and appreciate them; and in this the last charge I shall ever give you, I urge you to regulate your intercourse with your professional brethren upon the strictest principles of medical etiquette. There is another usage of medical men, which is exceedingly important, and for the practice of which the public are much more indebted to them than they appear willing to acknowledge. I allude to the custom among respectable physicians and surgeons to make known as widely as possible any discovery or improvement they may make in medicine. What knowledge of medicine we have is chiefly that we have received from those who have gone before us; and it is doubtlesss the duty of every medical man to add what he can to the common stock of knowledge. It is by the observance of this rule that medical science is daily expanding and improving. It is by self-denial, amounting in some instances to heroism, that the public are indebted for the cure and prevention of many fearful disorders. Yet how ungrateful are the public for the sacrifices thus made! What an immense mine of wealth was Jenner's discovery of vaccination, if he had kept his secret! He could have drawn to himself almost any portion he might have asked of the wealth of Europe. Kings would have knealt at his feet and besought protection from the frightful malady that mocked their greatness; and the rich would have poured out their treasures for the priceless secret. But what would the world have lost by his selfishness, if Jenner had considered the discovery as his property. I might mention other instances of the kind, that would show the immense obligation under which the public are placed by this noble rule of professional conduct. But how have they behaved ? Have they rewarded and reciprocated the generosity of physicians ? No, indeed. On the contrary, they offer premiums to secret nostrum-mongers. They reward by their confidence and their money those who are continually abusing and throwing contempt upon medical men. They will pour thousands into the lap of a candy maker, and do all they can to insult and injure their real benefactors. I own, gentlemen, that this conduct is well calculated to drive physicians from the honorable stand they have taken, and to make them secret mongers too. But I trust it will never be so. If the public do wrong, let us do right,—our duty is plain—and let us remember that he who hesitates between duty and inclination is undone.

There is still another usage of physicians which I would that you should imitate. It is our custom to decline receiving any compensation for any professional services we may render to one another, and to Ministers of the Gospel. With regard to the first, it is founded upon the spirit of brotherhood, and is a part of the system of reciprocity of which I have spoken. The second is a mark of respect to Clergymen—but is far from being gratuitous. The Clergyman is every man's servant. His time is appropriated by any one who may wish his counsel or instruction. Without fee or reward, he is required to do a great deal of arduous and unpleasant labor. It is but right, therefore, as he is always ready to visit us in our afflictions, we should be equally willing to minister to him.

There are many other things of which it would have afforded me pleasure to have spoken ; but as I cannot do so at length, I will detain you but a moment longer.

You are about to go forth to practice a useful art. Let it be your study to practice it in the spirit of benevolence. Go about doing good—alleviating suffering, and restoring health to as many as you can. Be glad to distribute of the good that you possess—a possession far more valuable than wealth, as it will enable you to promote the happiness of your fellow men, more certainly and in a greater measure than riches could do.

And, gentlemen, remember that for the proper use of your abilities and opportunities, you are directly responsible to God. *He* has never alienated from himself what he has bestowed upon you. He is the owner of your time and talent—your reason and your information. It is a fearful mistake to suppose that none are the stewards of the Almighty beneficence but those whom he has entrusted with money. There are powers, in comparison with which gold is insignificant, and with regard to all these the great Creator permits us to occupy only until He shall come to judge of our fidelity. Whatever we do, let us do it as unto Him—looking for his approbation, and walking in His precepts.

The profession you have chosen is not to be regarded merely as the means of your employment and creditable subsistence. It is to be the means of your mental and moral discipline—the mode of your probation—the form of your accountability. If you regard it as a mere worldly occupation, and enter upon it in the spirit of fallen self-love which men call selfishness, and which is the great antagonist sin, that everywhere opposes itself to Divine Benevolence. You will find it a pursuit full of vexation and disappointment; and in the eve of your lives will look back upon it with dissatisfaction and disgust, as a successful fraud upon your happiness. But if you enter upon it with cheerfulness as the way in which you are to employ your talents and your time—under the direction of God, to the good of yourselves and others, and especially to the glory of Him to whom time and talents belong—you will not fail to find in the practice of your profession a fund of enjoyment which will increase with the effort of every day.

Gentlemen, we now must take our leave of you. We cannot accompany you in your struggle with the difficulties and dangers of life. In your prosperity, we shall not be near you to warn you against presumption; and in adversity, we shall not be at hand to comfort and sustain you. A thousand circumstances necessarily limit the power of friendship—and render human sympathy valueless.

But in parting from you, we would affectionately remind you that there is a counsellor and a friend whose presence and whose wisdom will never fail you. To him we fervently commend you; and with our parting words we urge you to make his word your closest and most intimate companion. You will find in it all that you can ever want of advice and consolation. It will be a refuge in trouble, a shield in defence—an unfailing counsellor, and a sure guide to happiness!

Gentlemen, I bid you farewell.

---

**We hope that no one of our subscribers will lose any time, after the arrival of this number, before he reads with attention the excellent, or rather super-excellent, valedictory address, by Professor Bond, of Baltimore, to the graduates of the "Baltimore College of Dental Surgery," at the termination of the courses of Lectures last winter. The practical utility of such a production, can never be limited to the class to which it was first communicated; but its influence will be important and salutary on every well disposed an intelligent mind in our profession. Dr. Bond is not indeed a dentist; but as one of the Professors in the only Dental College in existence in this or any other country, his philosophic views of professional character and duties, will be universally gratifying to the profession, to the usefulness of which he has consented to devote a portion of his time and talent. If his other lectures of the course partook of the strongly marked talent, so manifest in this Valedictory, and if his associate professors manifest a corresponding zeal and ability, it cannot be denied by the most fastidious among the opposers of Dental Societies, Magazines and Colleges, that the young gentlemen who enjoy the advantages of such instruction as that given at this institution, have occasion to congratulate themselves on their peculiar good fortune. S. B.**

