# Errata

**Published:** 1841

**Authors:** 


### ERRATA.

Page 242, third line from bottom, for "*fitful,*" read "*pitiful.*"

" Next line, for "*an* infant enterprize," read "*our* infant enterprize."

" 243, about the middle of the page, for "*inquiring,*" read "*inquiry.*"

" 244, 7th line, for "*and* bound up, &c" read "*are* bound," &c.

" 244, last line, for "*management,*" read "*arrangement.*"

" 246, 7th line, for "*disperse*" read "*disprove,*" 10th line, for "*sensible*" read "*sensibility.*" 36th line, for "*wiser*" read "*wise.*"

" 248, 16th line, 2d paragraph for "if they are *not* natural," read "if they *are* natural."

" 250, for "Anarcharsis *Clooty,*" read "Anarcharsis *Clootz.*"

" 252, 4th paragraph, for "*secreting,*" read "*scrutiny.*" 254, 11th line, for "*increased,*" read "*inverse.*" 257, 2d line, a period is misplaced.

